# Oxygen-binding and sensing proteins

**DOI:** 10.12688/f1000research.6017.1

**Published:** 2015-01-09

**Authors:** Robert K. Poole

**Affiliations:** 1 Department of Molecular Biology and Biotechnology, University of Sheffield, Sheffield, S102TN, UK

**Keywords:** oxygen-binding protein, heme, globin, sensing protein

## Abstract

This collection of papers is a snapshot of topics presented at the international conference on *Oxygen-Binding and Sensing Proteins *(O2BiP) **held in Sheffield, UK, on 6 ^th^-10 ^th^ July 2014. The collection will grow over time as new papers relating to O2BiP topics are completed and published.

## Editorial

The meeting held in Sheffield, UK, on 6 ^th^–10 ^th^ July 2014 was the 18 ^th^ on the topic of oxygen-binding and sensing proteins (O2BiP). Previous meetings were held in Parma (Italy, 2012) and previously in Aarhus, Naples etc. This meeting has never been held in the UK, despite a long tradition of top-flight research in ligand binding to heme proteins, oxidative metabolism, bioenergetics and protein biochemistry. It is particularly appropriate that the meeting should be held in Sheffield because of a rich tradition of world-class research in the area of oxidative biochemistry, bioenergetics, and ligand-heme protein interactions. Among the luminaries who have worked in Sheffield are Lord Porter, Sir Hans Krebs, Gregorio Weber, Vincent Massey and Quentin Gibson.

Oxygen-binding proteins are arguably the most intensively studied of all proteins ^1^. The field has been revolutionised in recent years by paradigm shifts in our understanding of these proteins. Among these developments are (i) the discovery of globins in numerous animal species, plants and microbes, with important implications for globin evolution ^2, 3^, (ii) the recognition that the ligand binding and catalytic capability of ‘oxygen-binding proteins’ actually embraces physiologically critical ligands such as nitric oxide and hydrogen sulfide ^4, 5^, (iii) the sheer number of globins encoded by simple genomes ^6^, and (iv) the multiple roles of oxygen-binding proteins in natural environments and disease.

These meetings have always emphasized both the structure and function of globins and oxygen-related proteins at all levels of organisation from the molecular and cellular to the whole organism. These meetings have also demonstrated a remarkably multidisciplinary approach to the area, with contributions extending from molecular biophysics, to biochemistry and physiology and whole organism functions. Although primarily an experimental subject area, newer computational tools have proved important in evolutionary studies and modelling of ligand-protein interactions. The subject has considerable potential for contributing to knowledge that can be applied to the development of agriculture and medicine and to understanding the impacts of human activity on living organisms and ecosystems.

The collection of papers here is merely a snapshot of a remarkably diverse and exciting meeting. It included more than 50 lectures and 30 posters. The organisers of this O2BiP were anxious to involve younger, early career scientists in the meeting and some of these contributions can be seen in this collection.
